# Bidirectional association between falls and multimorbidity in middle-aged and elderly Chinese adults: a national longitudinal study

**DOI:** 10.1038/s41598-024-59865-z

**Published:** 2024-04-20

**Authors:** Ye Tian, Xingzhao Zhou, Yan Jiang, Yidan Pan, Xuefeidan Liu, Xingbo Gu

**Affiliations:** 1 Department of Health Statistics, School of Public Health, Hainan Medical University, No. 3, Xue Yuan Road, Longhua District, Haikou, 571199 People’s Republic of China; 2 Department of Marine Pharmacy, School of Pharmacy, Hainan Medical University, No. 3, Xue Yuan Road, Longhua District, Haikou, 571199 People’s Republic of China

**Keywords:** Multimorbidity, Falls, Bidirectional association, CHARLS, Risk factors, Epidemiology

## Abstract

This study explores the bidirectional association between multimorbidity and falls in Chinese middle-aged and elderly adults. Participants aged 45 and above from the China Health and Retirement Longitudinal Study were included. Binary logistic regression assessed the impact of chronic conditions on fall incidence (stage I), while multinomial logistic regression examined the relationship between baseline falls and multimorbidity (stage II). The fully adjusted odds ratios (ORs) for one, two, or three or more chronic conditions were 1.34, 1.65, and 2.02, respectively. Among participants without baseline falls, 28.61% developed two or more chronic conditions during follow-up, compared to 37.4% of those with a history of falls. Fully adjusted ORs for one, two, or three or more chronic conditions in those with a history of falls were 1.21, 1.38 and 1.70, respectively. The bidirectional relationship held in sensitivity and subgroup analyses. A bidirectional relationship exists between multimorbidity and falls in Chinese middle-aged and elderly adults. Strengthening chronic condition screening and treatment in primary healthcare may reduce falls risk, and prioritizing fall prevention and intervention in daily life is recommended.

## Introduction

The progress of modern medicine and the widespread dissemination of healthcare knowledge have brought about an increasingly pressing issue: the aging of the population, which has resulted in a significant rise in the prevalence of chronic conditions^1,2^. Moreover, due to the fact that a majority of middle-aged and elderly individuals are afflicted with more than one chronic condition simultaneously, the problem of multimorbidity in this population has become more salient^3^. Multimorbidity, defined as the co-occurrence of two or more chronic conditions, encompasses physical diseases, geriatric syndromes, and mental health issues^4,5^. Data from the China Health and Retirement Longitudinal Study reveals that the prevalence of multimorbidity among Chinese individuals aged 45 and above is 55.77%^5^. Multimorbidity poses significant risks to individuals, including system-function decline, multiple drug use, disability, and death, and places an onerous burden on the healthcare system^6–8^. Therefore, identifying and comprehending the risk factors of multimorbidity is of paramount importance in its prevention and control, and can provide a reference for the early monitoring and intervention needed to safeguard the physical health of the elderly.

The widely accepted definition of a fall entails a sudden, unintentional change in position causing an individual to land at a lower level, on an object, the floor, or the ground, other than as a consequence of sudden onset of paralysis, epileptic seizure, or overwhelming external force^9^. Falls represent a leading cause of severe adverse events, chronic conditions, and mortality among middle-aged and elderly individuals^10–12^. According to data from the World Health Organization (WHO), falls are the second most frequent cause of unintentional injury deaths globally, with an estimated 37.3 million severe fall-related injuries requiring medical attention each year^13^. Fatal fall-related injuries disproportionately affect middle-aged and older adults^14^. In China, approximately 50 million elderly people are estimated to experience at least one fall per year, with 36–44% of patients requiring emergency medical treatment post-fall^15^. Thereafter, patients may experience recurrent depression, disability, loss of independence, social isolation, or even death within a year^16,17^. Therefore, recognizing the key factors contributing to falls is of paramount significance in enhancing the health status of middle-aged and elderly individuals.

The co-occurrence of falls and multimorbidity is a frequent phenomenon among middle-aged and elderly adults. Previous studies have extensively reported the association between falls and specific chronic diseases or multimorbidity. The presence of chronic conditions in older adults has been shown to increase the likelihood of falls. For example, diabetes can cause neuropathy, impairing balance and heightening the risk of falls^18^. Prospective studies have shown that the occurrence of falls in middle-aged and elderly people with chronic conditions is at least 30% higher than that in people without chronic conditions at baseline, with the risk further increasing as the number of chronic conditions increases^19^. In contrast, there is limited direct evidence demonstrating that falls augment the incidence of comorbid chronic diseases. Nevertheless, evidence suggests that falls themselves can cause traumatic injuries, including fractures and sprains, while also potentially impacting the physical, psychological, immune functions and quality of life of this population, leading to long-term health problems^20–22^. For instance, falls can reduce physical activity levels in this population, increasing the likelihood of obesity, diabetes, and cardiovascular diseases^23^. Therefore, we hypothesize that the relationship between falls and comorbid chronic diseases may not be unidirectional, and a bidirectional association between them may exist. To our knowledge, no longitudinal cohort studies have examined the bidirectional relationship between falls and multimorbidity.

The aim of this study was to investigate the bidirectional relationship between multimorbidity and falls in Chinese adults aged 45 or older, using data from the China Health and Retirement Longitudinal Study (CHARLS). Firstly, we validated the longitudinal relationship between baseline multimorbidity and subsequent falls, a relationship that, despite being widely documented, still requires rigorous longitudinal evidence in the Chinese context. Secondly, in a more innovative approach, we investigated whether individuals who experienced falls are at an increased risk of developing multimorbidity over time. This dual focus not only aids in a more comprehensive understanding of the potential interactions between falls and chronic diseases but also emphasizes the importance of considering falls both as a consequence and a potential precursor of multimorbidity in the elderly.

## Methods

### Study population

The China Health and Retirement Longitudinal Study (CHARLS) is a comprehensive interdisciplinary survey project led by the National School of Development at Peking University and executed by the China Social Science Survey Center, which has received funding from the National Natural Science Foundation of China. The primary objective of CHARLS is to compile a high-quality dataset that represents households and individuals aged 45 years or older in China, for the purpose of analyzing population aging issues and promoting interdisciplinary research on aging^24^. This dataset provides a more rigorous basis for the formulation and improvement of relevant policies. The study follows a baseline sample tracking research model, which involves a nationwide baseline survey in 2011 and subsequent biennial follow-ups. The academic community gains access to the findings one year after the survey’s completion. To date, the study has amassed 4 waves of data, collected in 2011, 2013, 2015, and 2018.

**Figure 1:**
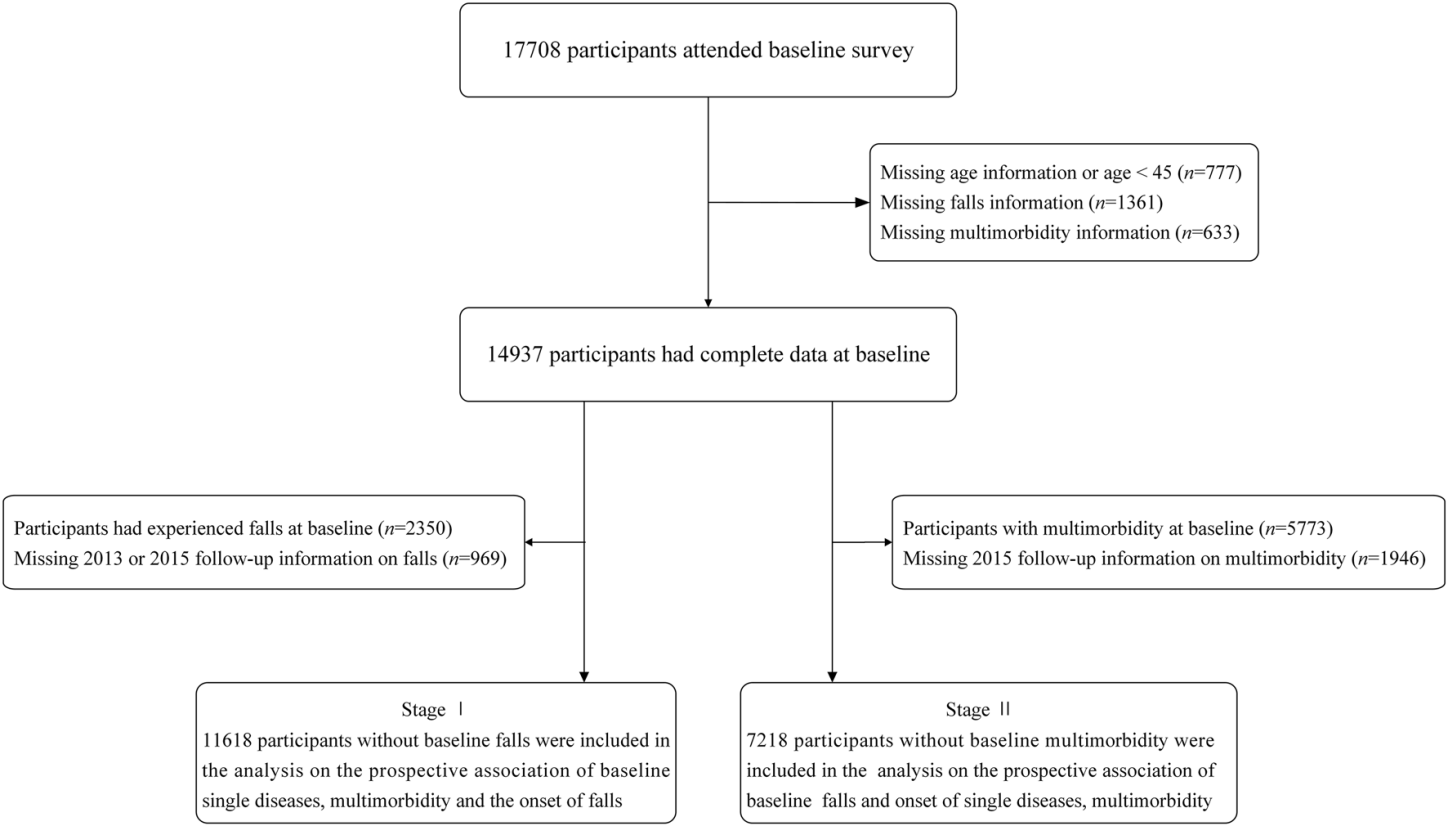
Flow chart for study population selection.

The present study represents a secondary analysis of data from the CHARLS, spanning from 2011 to 2015. In Stage I, 11,618 respondents without a history of falls at baseline were selected for inclusion, as depicted in detail in Fig. 1. In Stage II, a total of 7218 respondents without multimorbidity at baseline were included in the analysis. The CHARLS protocol had received permission from the Biomedical Ethics Committee of Peking University, and all participants provided informed consent at the time of participation.

### Assessment of chronic conditions and multimorbidity

In the CHARLS questionnaire, participants were asked if they had been diagnosed with any of the following 14 chronic conditions by a doctor: hypertension, diabetes, cancer, pulmonary disease, heart disease, stroke, mental illness (emotional, nervous, or psychiatric problems), arthritis, dyslipidemia, liver disease, kidney disease, gastrointestinal disease, asthma, and memory-related diseases (Alzheimer's disease, Parkinson's disease, and cerebral atrophy). All diseases were treated as binary variables. Multimorbidity was defined as the coexistence of two or more chronic diseases, and we further categorized based on the number of chronic conditions as: no chronic condition, one chronic condition, two chronic conditions, and three or more chronic conditions.

### Assessment of falls

In this study, falls was defined as a binary variable, with 0 indicating no fall and 1 indicating fall. In 2011, at baseline, participants were asked whether they had experienced any falls in the previous two years. The answer options were limited to a binary choice, either “yes” or “no”. In the subsequent two follow-ups (in 2013 and 2015), participants were asked about any fall events that occurred since their last interview. Similarly, the answer options were limited to a binary choice, either “yes” or “no”.

### Covariates

The selection of covariates in this study was informed by prior studies and based on data from the 2011 baseline survey. Covariates included age, gender, educational level (uncompleted primary school, primary school, middle school, or higher), marital status (married or cohabiting vs. separated, divorced, widowed, or never married), residential area (urban vs. rural, the definition of urban and rural areas is based on the classification by the National Bureau of Statistics of China, with communities or villages as the fundamental sampling units^25^), alcohol drinking (ever drinking vs. never drinking), smoking (ever smoking vs. never smoking), working status (currently working or not), Body Mass Index (BMI) grouped into four categories (< 18.5 kg/m^2^, 18.5–25 kg/m^2^, 25–30 kg/m^2^, and ≥ 30 kg/m^2^), and Activities of Daily Living(ADLs) limitation (participants were evaluated based on self-reported difficulty in dressing, bathing/showering, eating, getting in or out of bed, using the toilet, and controlling urination and defecation, with ADLs limitation represented as a binary variable reflecting difficulties in at least one of these activities^26^).

### Statistical analysis

Baseline characteristics were presented for both quantitative and qualitative variables. Means and standard deviations were calculated for quantitative variables, and independent two-sample *t*-tests or variance analysis were performed to compare baseline groups. Frequencies and percentages were used for qualitative variables, and chi-square tests were used to compare baseline groups. In Stage I, binary logistic regression models were employed to investigate the relationship between the baseline presence of multimorbidity and follow-up onset of falls. In Stage II, multinomial logistic regression models were used to estimate the odds ratio (OR) and 95% confidence interval (CI) for the association between falls and follow-up multimorbidity. Two models were established for the main analysis: the original model (Model 1) and a multivariate adjustment model adjusted for age, gender, BMI, education level, marital status, smoking, alcohol consumption, residential status, current employment status, and ADLs limitation (Model 2). During the data processing, we discovered that some selected covariates had missing values, but the amount of missing data remained within the acceptable range for statistical analysis (Supplementary Table 1). We addressed the issue of missing data during data processing by using the multiple imputation by chained equations (MICE) method based on random forest^27^. Five imputed datasets were created using the “mice” package in R, including all variables used in the analysis. Sensitivity analyses were conducted to evaluate the robustness of the results^28^. First, we conducted an analysis of the data before imputation to assess the impact of data imputation on the results. Second, we redefined multimorbidity into five levels (no chronic condition, one chronic condition, two chronic conditions, three chronic conditions and four or more chronic conditions) to evaluate the bidirectional relationship between falls and multimorbidity. Finally, subgroup analyses were conducted on age, gender, smoking, alcohol drinking, residential area, and working status (adjusted for the same covariates as the main analysis). All analyses were conducted using R 4.2.2, and a two-tailed *P* < 0.05 was considered statistically significant.

## Results

### Stage I: longitudinal association between baseline multimorbidity and incident falls during follow-up

Table 1 presents the baseline characteristics of the participants in Stage I of the survey who had no history of falls. Of the 11,618 participants, 33.3% had no chronic conditions, 30.2% had one chronic condition, 19.7% had two chronic conditions, and 9.6% had three or more chronic conditions. The participants with multiple chronic conditions at baseline were more likely to be older, female, urban residents, have lower levels of education, unmarried or have other marital statuses, have a history of smoking and alcohol consumption, be unemployed, and have higher levels of BMI and ADLs limitation than the group without chronic conditions at baseline. The risk of incident falls during follow-up significantly increased with the number of chronic conditions at baseline. After adjusting for confounding variables, including age, gender, BMI, smoking, alcohol drinking, education level, marital status, residential area, working status, and ADLs limitation, the adjusted ORs (95% CIs) were 1.34 (1.19, 1.51), 1.65 (1.45, 1.88), and 2.02 (1.77, 2.31) for one, two, and three or more chronic conditions, respectively. Thus, there is a significant dose–response relationship between the number of chronic conditions at baseline and incident falls during follow-up, as demonstrated by both unadjusted and adjusted models (*P* for trend < 0.001, Table 2). Additionally, baseline multimorbidity (Overall ≥ 2 chronic conditions) was significantly associated with falls during follow-up, with an adjusted ORs (95% CIs) was 1.55 (1.42, 1.70).

**Table 1:** Baseline characteristics of participants by number of chronic conditions.

Characteristics	All (*N* = 11,618)	Number of chronic conditions at baseline				*P*
		0 (*N* = 3867)	1 (*N* = 3505)	2 (*N* = 2284)	≥ 3 (*N* = 1111)	
Age	58.6 (9.36)	56.9 (9.30)	58.5 (9.33)	59.6 (9.28)	60.8 (8.97)	< 0.001
Gender (men)	5693 (49.0%)	2002 (51.8%)	1720 (49.1%)	1083 (47.4%)	888 (45.3%)	< 0.001
Education level						< 0.001
Not finish primary school	5055 (43.5%)	1570 (40.6%)	1575 (44.9%)	1032 (45.2%)	878 (44.8%)	
Sishu/elementary school	2567 (22.1%)	828 (21.4%)	748 (21.3%)	528 (23.1%)	463 (23.6%)	
Middle school and above	3996 (34.4%)	1469 (38.0%)	1182 (33.7%)	724 (31.7%)	621 (31.7%)	
Marital status						< 0.001
Married	10,254 (88.3%)	3469 (89.7%)	3097 (88.4%)	2000 (87.6%)	1688 (86.0%)	
Other	1364 (11.7%)	398 (10.3%)	408 (11.6%)	284 (12.4%)	274 (14.0%)	
Residential area						< 0.001
Urban	4598 (39.6%)	1461 (37.8%)	1361 (38.8%)	912 (39.9%)	864 (44.0%)	
Rural	7020 (60.4%)	2406 (62.2%)	2144 (61.2%)	1372 (60.1%)	1098 (56.0%)	
Ever alcohol drinking	4722 (40.7%)	1641 (42.5%)	1393 (39.8%)	916 (40.1%)	772 (39.4%)	0.047
Ever smoking	4630 (39.9%)	1590 (41.1%)	1392 (39.7%)	888 (38.9%)	760 (38.8%)	0.209
Working status	7334 (64.2%)	2728 (71.7%)	2309 (67.1%)	1342 (59.8%)	955 (49.6%)	< 0.001
BMI						< 0.001
< 18.5	634 (6.5%)	203 (6.2%)	187 (6.3%)	142 (7.3%)	102 (6.4%)	
18.5–25	6130 (62.7%)	2238 (68.4%)	1921 (64.9%)	1167 (59.8%)	804 (50.3%)	
25–30	2539 (26.0%)	721 (22.0%)	729 (24.6%)	535 (27.4%)	554 (34.7%)	
≥ 30	476 (4.9%)	108 (3.3%)	122 (4.1%)	109 (5.6%)	137 (8.6%)	
ADLs limitation	1561 (13.6%)	252 (6.61%)	404 (11.7%)	380 (16.8%)	525 (26.9%)	< 0.001
Falls (wave 3)	2680 (23.1%)	657 (17.0%)	785 (22.4%)	613 (26.8%)	625 (31.9%)	< 0.001

*BMI* body mass index.

**Table 2:** Longitudinal association between single disease, multimorbidity and falls.

Number of chronic conditions	Cases/No. (%)	Model 1	Model 2
0	657/3867 (17.0%)	Reference	Reference
1	785/3505 (22.4%)	1.41 (1.26, 1.58), < 0.001	1.34 (1.19, 1.51), < 0.001
2	613/2284 (26.8%)	1.79 (1.58, 2.03), < 0.001	1.65 (1.45, 1.88), < 0.001
≥ 3	625/1962 (31.9%)	2.28 (2.01, 2.59), < 0.001	2.02 (1.77, 2.31), < 0.001
*P* for trend		< 0.001	< 0.001
Overall (≥ 2)	1238/4246 (29.2%)	1.69 (1.55, 1.85), < 0.001	1.55 (1.42, 1.70), < 0.001

Data are presented as odds ratio (95% confidence interval) and *P* value.

Model 1: Crude.

Model 2: Adjusted for age, gender, BMI, smoking, alcohol drinking, education, marital status, residential area, work status and ADLs limitation.

### Stage II: longitudinal association between baseline falls and incident multimorbidity during follow-up

Baseline characteristics of the study participants without multimorbidity at baseline, who were included in Stage II, are shown in Table 3. Among the 7218 participants surveyed at baseline, 949 (13.2%) had experienced falls. After two waves of follow-ups in 2013 and 2015, 28.6% of participants without falls and 37.4% of those with falls were diagnosed with multimorbidity. Those with falls at baseline were relatively older, more likely to be female, have lower levels of education, reside in rural areas, currently drink alcohol, and have ADLs limitation. Table 4 showed that compared to participants without falls at baseline, the adjusted ORs (95% CIs) for developing one, two, or three or more chronic conditions during follow-up for those with falls were 1.21 (0.99, 1.46), 1.38 (1.10, 1.72) and 1.70 (1.31, 2.21), respectively. And baseline falls was significantly associated with follow-up multimorbidity (Overall ≥ 2 chronic conditions), with an adjusted ORs (95% CIs) was 1.34 (1.15, 1.55).

**Table 3:** Baseline characteristics of participants by falls status.

Characteristics	All (*N* = 7218)	Falls		*P*
		No (*N* = 6269)	Yes (*N* = 949)	
Age	57.7 (9.15)	57.4 (9.11)	59.1 (9.28)	< 0.001
Gender (men)	3586 (49.7%)	3159 (50.4%)	427 (45.0%)	0.002
Education level				< 0.001
Did not finish primary school	3185 (44.1%)	2684 (42.8%)	501 (52.8%)	
Sishu/elementary school	1543 (21.4%)	1350 (21.5%)	193 (20.3%)	
Middle school and above	2490 (34.5%)	2235 (35.7%)	255 (26.9%)	
Marital status				0.062
Married	6443 (89.3%)	5613 (89.5%)	830 (87.5%)	
Other	775 (10.7%)	656 (10.5%)	119 (12.5%)	
Residential area				0.019
Urban	2624 (36.4%)	2312 (36.9%)	312 (32.9%)	
Rural	4594 (63.6%)	3957 (63.1%)	637 (67.1%)	
Ever alcohol drinking	3014(41.8%)	2580(41.2%)	434(45.8%)	0.008
Ever smoking	2909(40.3%)	2542(40.6%)	367(38.6)	0.262
Working status	5044 (70.9%)	4393 (71.2%)	651 (69.3%)	0.236
BMI				0.369
< 18.5	389 (6.28%)	326 (6.08%)	63 (7.6%)	
18.5–25	4170 (67.3%)	3615 (67.4%)	555 (66.7%)	
25–30	1406 (22.7%)	1225 (22.8%)	181 (21.8%)	
≥ 30	230 (3.7%)	197 (3.7%)	33 (4.0%)	
ADLs limitation	733 (10.3%)	536 (8.7%)	197 (21.0%)	< 0.001
Number of chronic conditions (wave3)				< 0.001
0	2152 (29.8%)	1928 (30.8%)	224 (23.6%)	
1	2916 (40.4%)	2546 (40.6%)	370 (39.0%)	
2	1418 (19.6%)	1199 (19.1%)	219 (23.1%)	
≥ 3	732 (10.1%)	596 (9.5%)	136 (14.3%)	
Multimorbidity (wave3)	2150 (29.8%)	1795 (28.6%)	355 (37.4%)	< 0.001

**Table 4:** Longitudinal association between falls and single disease, multimorbidity.

	Number of chronic conditions			
	1	2	≥ 3	Overall (≥ 2)
Cases/No. (%)	370/2916 (12.7%)	219/1418 (15.4%)	136/732 (18.6%)	355/2150 (16.5%)
Model 1	1.25 (1.05, 1.49), 0.013	1.57 (1.29, 1.92), < 0.001	1.96 (1.56, 2.48), < 0.001	1.49 (1.29, 1.72), < 0.001
Model 2	1.21 (0.99, 1.46), 0.058	1.38 (1.10, 1.72), 0.005	1.70 (1.31, 2.21), < 0.001	1.34 (1.15, 1.55), < 0.001

Data are presented as odds ratio (95% confidence interval) and *P* value.

Model 1: Crude.

Model 2: Adjusted for age, gender, BMI, smoking, alcohol drinking, education, marital status, residential area, work status and ADLs limitation.

### Sensitivity and subgroup analyses

In sensitivity analysis, analysis of unfilled data reveals that there is still a bidirectional association between multimorbidity and falls, with minimal changes in the values of odds ratios and 95% confidence intervals (Supplementary Tables 2 and 3). Similarly, even after redefining the main variables, the bidirectional association between multimorbidity and falls remains significant. As shown in Supplementary Table 4, when the multimorbidity were defined as 5 levels, the risk of falls during follow-up was significantly higher for participants with four or more baseline chronic conditions (adjusted OR of 2.18, 95% CI of 1.83–2.59). As shown in Supplementary Table 5, when the multimorbidity were defined as 5 levels, the risk of developing multiple chronic conditions during follow-up was still significantly higher for participants who had a history of falls at baseline, compared to those who did not, adjusted ORs (95% CIs) 1.21 (0.99, 1.46), 1.38 (1.10, 1.72), 1.62 (1.21, 2.16) and 1.95 (1.30, 2.95) for developing one, two, three, four or more chronic conditions during follow-up.

**Figure 2:**
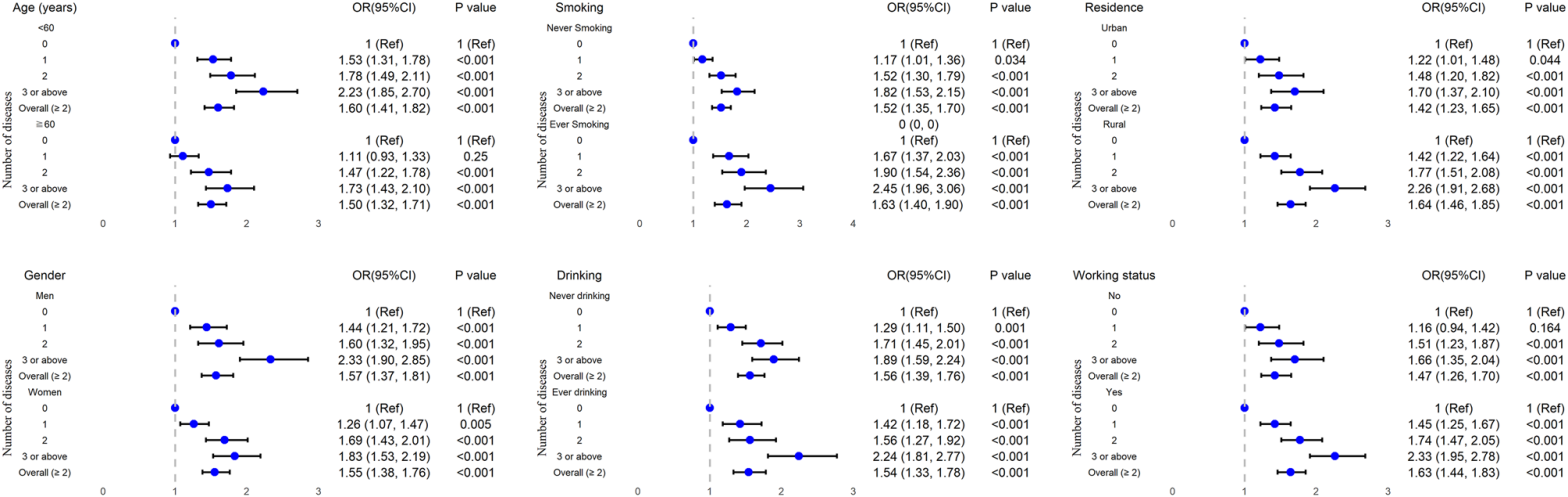
Subgroup analyses for the longitudinal association between single disease, multimorbidity and falls. Models adjusted for age, gender, smoking, alcohol drinking, residential area, and working status (excluding stratified variables).

**Figure 3:**
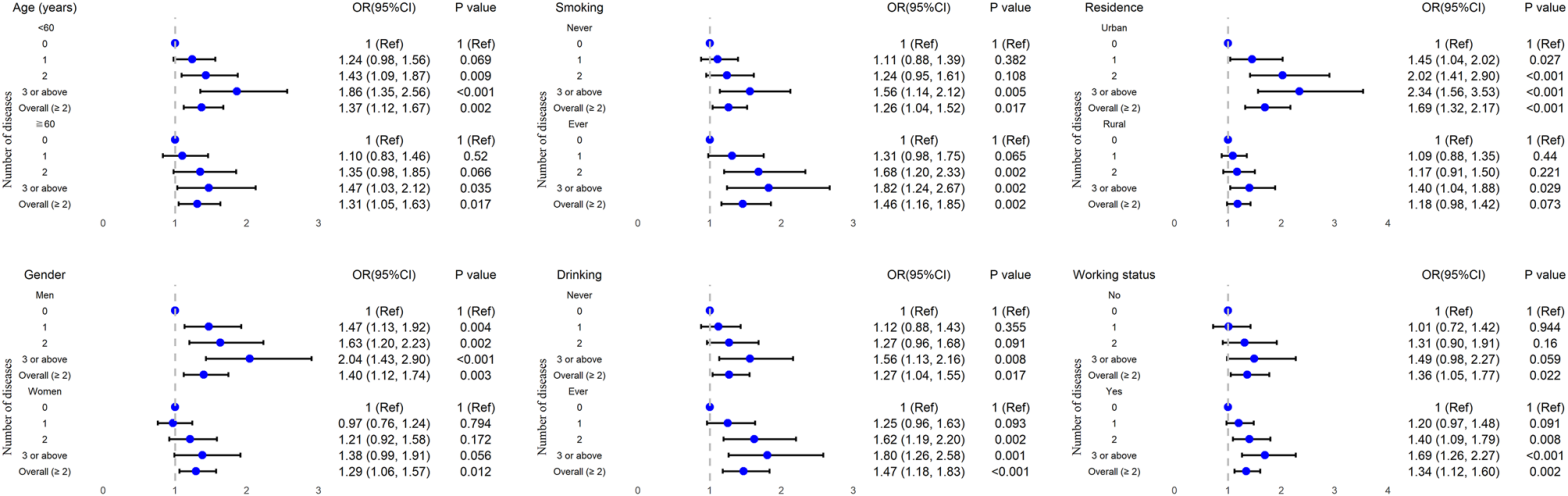
Subgroup analyses for the longitudinal association between falls and single disease, multimorbidity. Models adjusted for age, gender, smoking, alcohol drinking, residential area, and working status (excluding stratified variables).

In subgroup analysis stratified by age, gender, smoking, alcohol drinking, residential area and working status, no statistically significant interaction was found. The longitudinal relationship between baseline multimorbidity and new-onset falls during follow-up remained significant in almost all subgroups (Fig. 2). Contrary to expectations, although no statistically significant interaction was observed in the analysis of the longitudinal relationship between baseline falls and the onset of new multimorbidity during follow-up, this association persisted in most subgroups. Notably, in certain subgroups, such as residential status, a distinctive pattern emerged: baseline falls were found to elevate the risk of developing multimorbidity specifically in urban areas during the follow-up period, in contrast to rural areas (Fig. 3).

## Discussion

Drawing upon longitudinal data from the Chinese Health and Retirement Longitudinal Study, our study has uncovered a bidirectional relationship between multimorbidity and falls. The incidence of falls during follow-up significantly increases with an increase in the number of baseline chronic conditions, demonstrating a clear dose–response relationship. On the contrary, participants who reported falls at baseline were significantly more likely to develop multiple chronic conditions during follow-up. Even after adjusting for various demographic, lifestyle, and other factors and redefining the main study variables, our results were robust and stable.

To our knowledge, this study provides the first longitudinal evidence of the bidirectional association between multimorbidity and falls in middle-aged and older adults. Prior studies have largely focused on the independent association between specific chronic conditions or multimorbidity and falls. For instance, Beretta et al. reported that patients with type 2 diabetes and orthostatic hypotension had a 2.7-fold higher risk of in-hospital falls compared to those without these conditions^29^. Farrell et al. found that patients with a history of polypharmacy, chronic kidney disease, or malignancy were at an increased risk of adverse clinical outcomes following falls^30^. Sibley et al. surveyed over 15,000 community-dwelling older adults aged 65 and older in Canada and found that those with multiple chronic conditions were more likely to experience falls than those with only one chronic condition^31^. Similar to our study, Yan et al. also utilized CHARLS data and found an increased risk of falls associated with baseline multimorbidity: compared to patients without chronic conditions, those with one, two, and three or more chronic diseases had a 37%, 85%, and 175% increased risk of falls, respectively^32^. However, the core focus of Yan et al.'s study was on examining the relationship between four specific patterns of multimorbidity—cardiac-metabolic, visceral-arthritic, respiratory, and mental-sensory—identified using exploratory factor analysis (EFA)—and the risk of falls. Our study further validated the robustness of the association between various chronic conditions and the risk of falls through the use of multiple subgroup analyses and sensitivity analyses. Additionally, we proposed a finding that baseline falls could also lead to an increased risk of multimorbidity during follow-up. Prior to this, there has been a lack of high-quality direct evidence directly linking falls to an increased incidence of multimorbidity, with only indirect evidence suggesting a potential association^23,33,34^. Building upon this, our study proposes a bidirectional relationship between falls and multimorbidity. Additionally, our study found some noteworthy findings. For instance, subgroup analysis results indicate that the increased risk of developing multimorbidity after baseline falls was primarily observed in urban populations, with this association not being observed in rural populations. The increased risk of developing chronic comorbidities in urban populations after baseline falls may be partly explained by the complex environments and different lifestyle factors experienced by urban residents, where demanding physical and social infrastructure may exacerbate the impact of falls^35^. Additionally, urban residents generally have better access to healthcare resources compared to rural areas, potentially facilitating quicker post-fall treatment but also potentially increasing the identification and reporting of chronic comorbidities during the treatment process^36^.

Bidirectional associations between multimorbidity and falls may arise from various underlying mechanisms. Firstly, recurrent onset and treatment of chronic conditions may induce physical functional decline, which reduces muscle strength and overall vitality and renders multimorbid patients more susceptible to falls due to lower resistance capacity^37^. Additionally, multiple chronic conditions can result in pathological balance disorders, including brainstem dysfunction, nerve compression stemming from intervertebral disc degeneration, and osteoporosis, all of which can trigger falls. Common chronic ailments such as arteriosclerosis, hypertension, diabetes, respiratory and liver diseases may also lead to balance disorders^38^. The effects of drug side effects on walking and balance ability, such as motor inhibition induced by antiepileptic drugs or dizziness caused by antihypertensive drugs and certain neuroinhibitors, can also exacerbate the risk of falls^39,40^. Given that individuals with multimorbidity often require multiple medications, the impact of polypharmacy on them is particularly salient, and there is a strong correlation between polypharmacy and fall risk^41^. On the other hand, the mechanisms by which falls contribute to chronic conditions may involve the prolonged rest and recovery needed for soft tissue injuries and fractures caused by falls, which can lead to muscle atrophy and bone loss, especially among older adults, increasing the risk of developing chronic diseases^42^. This mechanism unveils how falls could directly lead to the development of new comorbidities. Further, falls may be an early indicator of cognitive decline and are linked to an increased risk of diseases like dementia; this connection was specifically investigated in a study by the Einstein Institute on Aging, which examined the relationship between falls, cognitive decline, and the risk of Motor Cognitive Risk (MCR) Syndrome and dementia^43^. The study revealed that among older adults, experiencing multiple falls is correlated with a more pronounced decline in overall cognitive abilities, including aspects such as global cognition, situational memory, verbal fluency, and processing speed-attention. This underscores the possibility that falls could act as early signs of potential undiagnosed conditions, such as cognitive decline and dementia. Moreover, falls can trigger negative psychological effects, such as fear, anxiety, and depression, which can impair the body's immune and metabolic functions, further amplifying the risk of developing chronic conditions^33^. This not only suggests that falls may lead to new comorbidities but also reflects the possibility that falls themselves could be a manifestation of underlying, yet undiagnosed health issues. In summary, fall events may serve as direct catalysts for the development of new comorbidities or as early indicators of undiagnosed conditions. These aspects underscore the vital importance of fall prevention and management in preserving the health of individuals with multimorbidity.

Our findings present some limitations. Firstly, the information on the 14 primary chronic conditions that constitute multimorbidity and falls was collected via self-reports. Although this method has been extensively used in other large-scale population-based studies and demonstrated good specificity and positive predictive value^44,45^, recall bias and reporting errors may still occur among middle-aged and elderly individuals. The follow-up period of this study spans approximately four years. During recall of events over this period, participants may inadvertently forget about non-fatal falls or overlook less severe chronic conditions. Secondly, while we attempted to incorporate potential covariates that may affect the study, certain covariates, such as physical activity level, were not included in the analysis due to insufficient reporting in the CHARLS database (missing over 40%). Similarly, it is crucial to acknowledge the importance of the number and frequency of falls in investigating the relationship between falls and multimorbidity. However, in the CHARLS dataset, the proportion of missing data related to fall frequency exceeded 80%, precluding further exploration of the association between fall frequency and multimorbidity. Thirdly, our study solely considered the quantity of chronic conditions (a widely utilized and straightforward approach) in the investigation of multimorbidity and falls, without further examining the impact of specific types of chronic conditions or combinations of chronic conditions on the study. Future study may explore the bidirectional relationship between different multimorbidity patterns and falls by clustering different chronic conditions based on their weight. Fourthly, recognizing falls as a time-varying variable indeed adds depth to our understanding of their associations. To precisely capture the nuances of this dynamic variable, the use of wearable devices, such as the Mobility Interaction Fall chart, employing inertial sensors, video/depth cameras, pressure sensing platforms, and laser sensors, could offer a more accurate assessment of daily falls^46^. However, constrained by the available CHARLS data, our analysis focused on falls reported during a single survey, limiting our ability to explore temporal changes comprehensively. Fifthly, it must be acknowledged that our study, which analyzes the bidirectional relationship between multimorbidity and falls within the CHARLS cohort, may encounter competing risks. However, CHARLS only released detailed information on deaths and withdrawals in wave 1 and wave 2, with subsequent waves lacking such data. Although the recently published wave 5 of CHARLS appears to include information about participant withdrawals, this wave was conducted during the COVID-19 pandemic and involved changes in survey content. Therefore, our study did not further explore potential competing analyses based on this latest data. Lastly, although the CHARLS is a representative nationwide cohort, the study population only encompasses middle-aged and elderly individuals in China. Caution must be exercised when generalizing the study's results to other ethnic groups.

## Conclusion

Utilizing the CHARLS dataset, this study reveals a bidirectional link between multimorbidity and falls in China's middle-aged and elderly, challenging the traditional view of falls merely leading to multimorbidity. It underscores the need for improved chronic disease management and fall prevention in primary care. This bidirectional emphasis encourages a holistic healthcare approach, integrating fall prevention and chronic disease management. Understanding this relationship's mechanisms and extending these insights to diverse populations is essential for future healthcare strategies.

### Supplementary Information

Supplementary Tables.

## Data Availability

The data that support the findings of this study are available on the website of the China Health and Retirement Longitudinal Study (CHARLS) at https://charls.charlsdata.com/pages/data/111/zh-cn.html. To access and use this survey data for research purpose, an approval should be obtained from the CHARLS team at Peking University. You could contact the corresponding author (hy0208051@hainmc.edu.cn) if you want to request the data from this study.

## Abbreviations

CHARLS: Chinese Health and Retirement Longitudinal Study
CIs: Confidence intervals
ORs: Odds ratios
BMI: Body mass index
ADLs: Activities of daily living
MICE: Multiple imputation by chained equations
