# Supplementary material for: Bidirectional association between falls and multimorbidity in middle-aged and elderly Chinese adults: a national longitudinal study
Source: Sci Rep. 2024 Apr 20;14:9109. doi: 10.1038/s41598-024-59865-z (PMC11032330; doi:10.1038/s41598-024-59865-z)
Supplement: Supplementary file 1 — Supplementary Tables. [file 41598_2024_59865_MOESM1_ESM.docx]

**Additional file 1**

**Bidirectional association between falls and multimorbidity in middle-aged and elderly Chinese adults: A national longitudinal study**

Ye Tian ^1†^, Xingzhao Zhou^1†^, Yan Jiang^1^, Yidan Pan^1^, Xuefeidan Liu^2^, Xingbo Gu^1*^

^1^ Department of Health Statistics, School of Public Health, Hainan Medical University, No. 3, Xue yuan Road, Longhua District, Haikou, 571199, P. R. China.

^2^ Department of Marine Pharmacy, School of Pharmacy, Hainan Medical University, No. 3, Xue yuan Road, Longhua District, Haikou, 571199, P. R. China.

Supplementary Table 1. Description of the missing data

| Characteristics | Stage Ⅰ (*N*=11618) |  | Stage Ⅱ (*N*=7218) |
| --- | --- | --- | --- |
| Age | 0 |  | 0 |
| Gender | 0 |  | 0 |
| Education level | 0 |  | 0 |
| Marital Status | 0 |  | 0 |
| Residential area | 0 |  | 0 |
| Alcohol drinking | 10 (0.08%) |  | 7 (0.09%) |
| Smoking | 7 (0.06%) |  | 5 (0.06%) |
| Working Status | 201 (1.72%) |  | 108 (1.48%) |
| BMI | 1839 (15.81%) |  | 1023 (14.17%) |
| Depression | 124 (1.06%) |  | 76 (1.04%) |
| ADLs limitation | 130 (1.12%) |  | 92 (1.27%) |

Supplementary Table 2. Sensitivity analyses for the longitudinal association between single disease, multimorbidity and falls before data imputation

| Number of chronic conditions | Cases/No.  (%) | Model 1 | Model 2 |
| --- | --- | --- | --- |
| 0 | 657/3867 (17.0%) | Reference | Reference |
| 1 | 785/3505  (22.4%) | 1.41 (1.26, 1.58),  <0.001 | 1.46 (1.28, 1.66),  <0.001 |
| 2 | 613/2284  (26.8%) | 1.79 (1.58, 2.03),  <0.001 | 1.78 (1.55, 2.05),  <0.001 |
| ≥3 | 625/1962  (31.9%) | 2.28 (2.01, 2.05),  <0.001 | 2.18 (1.88, 2.53),  <0.001 |
| *P* for trend |  | <0.001 | <0.001 |
| Overall (≥ 2) | 1238/4246 (29.2%) | 1.69 (1.55, 1.85),  <0.001 | 1.60 (1.45, 1.77),  <0.001 |

Data are presented as odds ratio (95% confidence interval) and *P* value.

Model 1: Crude.

Model 2: Adjusted for age, gender, BMI, smoking, alcohol drinking, education, marital status, residential area, work status and ADLs limitation.

Supplementary Table 3. Sensitivity analyses for the longitudinal association between falls and single disease, multimorbidity before data imputation

|  | Number of chronic conditions | | | |
| --- | --- | --- | --- | --- |
|  | 1 | 2 | ≥3 | Overall (≥ 2) |
| Cases/No.(%) | 370/2916 (12.7%) | 219/1418 (15.4%) | 136/732 (18.6%) | 355/2150 (16.5%) |
| Model 1 | 1.25 (1.05, 1.49),  0.013 | 1.57 (1.29, 1.92),  <0.001 | 1.96 (1.56, 2.48),  <0.001 | 1.49 (1.29, 1.72),  <0.001 |
| Model 2 | 1.21 (0.99, 1.46),  0.057 | 1.38 (1.10, 1.72),  0.005 | 1.70 (1.31, 2.21),  <0.001 | 1.32 (1.13, 1.55),  0.001 |

Data are presented as odds ratio (95% confidence interval) and *P* value.

Model 1: Crude.

Model 2: Adjusted for age, gender, BMI, smoking, alcohol drinking, education, marital status, residential area, work status and ADLs limitation.

Supplementary Table 4. Sensitivity analysis of the longitudinal association between single disease, multimorbidity and falls, where multimorbidity was redefined

| Number of chronic conditions | Cases/No. (%) | Model 1 | Model 2 |
| --- | --- | --- | --- |
| 0 | 657/3867  (17.0%) | Reference | Reference |
| 1 | 785/3505  (22.4%) | 1.41 (1.26, 1.58) ,  <0.001 | 1.34 (1.19, 1.51) ,  <0.001 |
| 2 | 613/2284  (26.8%) | 1.79 (1.58, 2.03),  <0.001 | 1.65 (1.46 , 1.88),  <0.001 |
| 3 | 337/1111  (30.3%) | 2.13 (1.83, 2.48),  <0.001 | 1.91 (1.63, 2.24),  <0.001 |
| ≥4 | 288/851  (33.8%) | 2.50 (2.12, 2.95),  <0.001 | 2.18 (1.83, 2.59),  <0.001 |
| *P* for trend |  | <0.001 | <0.001 |

Data are presented as odds ratio (95% confidence interval) and *P* value.

Model 1: Crude.

Model 2: Adjusted for age, gender, BMI, smoking, alcohol drinking, education, marital status, residential area, work status and ADLs limitation.

Supplementary Table 5. Sensitivity analyses for the longitudinal association between falls and single disease, multimorbidity, where multimorbidity was redefined

|  | Number of chronic conditions | | | | |
| --- | --- | --- | --- | --- | --- |
|  | 1 | 2 | 3 | ≥4 | Overall (≥ 2) |
| Cases/No. (%) | 370/2916 (12.7%) | 219/1418 (15.4%) | 92/525 (17.5%) | 44/207 (21.3%) | 355/2150 (16.5%) |
| Model 1 | 1.25 (1.05, 1.49),  0.013 | 1.57 (1.29, 1.92),  <0.001 | 1.83 (1.40, 2.38),  <0.001 | 2.32 (1.62, 3.33),  <0.001 | 1.49 (1.29, 1.72),  <0.001 |
| Model2 | 1.21 (0.99, 1.46),  0.058 | 1.38 (1.10, 1.72),  0.005 | 1.62 (1.21, 2.16),  0.001 | 1.95 (1.30, 2.95),  0.001 | 1.34 (1.15, 1.55),  0.001 |

Data are presented as odds ratio (95% confidence interval) and *P* value.

Model 1: Crude.

Model 2: Adjusted for age, gender, BMI, smoking, alcohol drinking, education, marital status, residential area, work status and ADLs limitation.
